# Clinical and Cosmetic Outcomes of Distal Resection Combined with Proximal Release in Children Older than 3 Years with Congenital Muscular Torticollis

**DOI:** 10.3390/children13050585

**Published:** 2026-04-23

**Authors:** Ahmet Yılmaz, Mehmet Yiğit Gökmen

**Affiliations:** 1 Department of Orthopaedics and Traumatology, Adana City Training and Research Hospital, University of Health Sciences, Adana 01230, Türkiye; 2 Department of Orthopaedics and Traumatology, Faculty of Medicine, Çanakkale Onsekiz Mart University, Çanakkale 17110, Türkiye; mehmet_yigit_gokmen@hotmail.com

**Keywords:** congenital muscular torticollis, sternocleidomastoid, bipolar release, cosmetic result

## Abstract

**Background**: Congenital muscular torticollis (CMT) is usually managed conservatively during infancy, whereas surgical treatment is considered for persistent deformity in older children. However, evidence remains limited regarding the outcomes of distal resection combined with proximal release of the sternocleidomastoid muscle in children presenting beyond infancy. This study aimed to evaluate the functional and cosmetic outcomes of this combined approach in patients aged 3 years and older. **Methods**: This retrospective single-surgeon series included 37 patients with CMT aged 3 to 14 years who underwent distal resection combined with proximal release of the sternocleidomastoid muscle between 2002 and 2024. Preoperative and postoperative assessments were performed using the clinical outcome framework originally described by Lee et al., goniometric measurement of cervical rotation and lateral flexion, and clinical evaluation of head tilt, facial asymmetry, scar appearance, lateral band formation, and sternocleidomastoid V-column contour. Patients were also analyzed according to age at surgery, as 3–10 years and 11–14 years. **Results**: The mean age at surgery was 4.7 years, and the mean follow-up duration was 3.4 years. Significant postoperative improvement was observed in all major functional outcomes. Mean cervical rotation improved from 54.2 ± 8.6° to 87.9 ± 3.4°, and mean lateral flexion improved from 24.1 ± 6.8° to 44.5 ± 3.2° (both *p* < 0.001). Preoperative functional assessment scores averaged 6.8 ± 1.4, whereas postoperative total outcome scores averaged 14.2 ± 0.9. At final follow-up, no patient had residual head tilt. Mild residual facial asymmetry persisted in 3 patients (8.1%). Overall, postoperative outcomes were rated as excellent in 33 patients (89.2%) and good in 4 patients (10.8%). A slight partial loss of the sternocleidomastoid V-column contour was observed in 34 patients (91.9%), although this finding was not documented as a major cosmetic concern in the available clinical records. Hypertrophic scarring developed in 1 patient (2.7%). No lateral band formation, recurrence, revision surgery, infection, or hematoma was observed. **Conclusions**: Distal resection combined with proximal release provided favorable functional and cosmetic outcomes in children older than 3 years with CMT. The technique was associated with marked improvement in cervical motion, correction of head tilt, low complication rates, and a high proportion of excellent or good results.

## 1. Introduction

Congenital muscular torticollis (CMT) is a postural deformity caused by unilateral shortening and fibrosis of the sternocleidomastoid (SCM) muscle, resulting in ipsilateral head tilt and contralateral chin rotation. Its reported incidence ranges from 0.3% to 1.9%, and it is considered the third most common congenital musculoskeletal deformity after pes equinovarus and developmental dysplasia of the hip. Because CMT frequently coexists with developmental dysplasia of the hip, careful musculoskeletal assessment is recommended in affected infants and children [1,2,3]. Although CMT has long been recognized, its etiology remains incompletely understood. Several mechanisms have been proposed, including birth-related trauma, intrauterine malposition, venous outflow obstruction, and ischemic injury of the SCM muscle, all of which may contribute to intramuscular fibrosis and subsequent contracture. Breech presentation and difficult delivery have been suggested as possible contributing factors. It has also been proposed that intrauterine malposition of the SCM muscle may produce a compartment-like effect, ultimately leading to fibrosis and contracture [3,4,5]. Because torticollis may also arise from nonmuscular causes, the differential diagnosis should include cervical vertebral anomalies, ocular disorders, neurologic conditions, hearing-related postural imbalance, infection, inflammation of adjacent tissues, and posttraumatic deformities [6,7].

Most patients with CMT are treated conservatively during infancy, primarily with stretching-based physiotherapy programs aimed at reversing SCM tightness and restoring cervical motion [8,9]. Early treatment is associated with the highest success rates [1,3,8,9]. Recent evidence syntheses have further supported the effectiveness of early physiotherapy and non-surgical treatment in appropriately selected infants, while also reinforcing the role of surgery in children with persistent deformity or failed conservative management [10,11]. In patients who present early but fail to improve with conservative treatment, operative treatment is generally considered after 1 year of age, with many reports describing favorable outcomes when surgery is performed between 1 and 4 years of age [6,12]. When diagnosis or referral is delayed, progressive secondary changes may develop, including persistent head tilt, compensatory facial rotation, ipsilateral shoulder elevation, craniofacial asymmetry, and plagiocephaly. In such cases, surgical treatment is often considered when deformity and limitation of motion persist beyond the usual period of effective conservative management [1,3,8,13].

Several surgical techniques have been described for patients who require operative treatment, including distal unipolar release, bipolar release, Z-plasty lengthening, partial muscle resection, and endoscopic approaches [12,14,15,16]. Despite the variety of available techniques, the optimal surgical strategy in children presenting beyond infancy remains debated. Significant scar formation, lateral band formation, loss of the SCM V-column appearance, and recurrence have been reported after some distal release or distal partial resection procedures. Bipolar release is often preferred in older children or in cases with more established deformity, but distal resection combined with proximal release has been reported less frequently and remains less well characterized in the literature [17,18,19]. In addition, contemporary studies have continued to explore alternative surgical strategies and patient-reported outcomes, highlighting that the choice of procedure remains clinically relevant and not yet fully standardized [20,21].

Accordingly, there remains limited evidence regarding whether distal resection combined with proximal release can provide satisfactory correction while minimizing unfavorable cosmetic and functional sequelae in children older than 3 years.

In this study, we evaluated the clinical and cosmetic outcomes of distal resection combined with proximal release of the SCM muscle in children with CMT aged 3 years and older. Specifically, we aimed to determine whether this combined approach could achieve effective deformity correction with a low rate of postoperative complications in patients presenting beyond infancy.

## 2. Materials and Methods

### 2.1. Ethical Approval

Ethical approval for this retrospective study was obtained from the Scientific Research Ethics Committee of the Adana City Training and Research Hospital (Meeting No: 23, Date: 25 February 2026, Decision No: 1221). Because of the retrospective design, the requirement for study-specific informed consent for retrospective data use was waived by the ethics committee. Written surgical informed consent had been obtained from the parents or legal guardians of all children before the operative procedure. All clinical data were evaluated in accordance with ethical standards.

### 2.2. Study Design and Participants

This study was designed as a retrospective observational study. A total of 37 patients aged 3 years and older who underwent distal resection and proximal release of the sternocleidomastoid muscle for congenital muscular torticollis (CMT) between 2002 and 2024 were evaluated. Among the patients operated on during the study period, 37 met the inclusion criteria and had complete clinical follow-up data available for review. There were 21 girls and 16 boys, with a mean age of 4.7 years (range, 3 to 14 years). Of these, 31 patients (83.8%) were aged 3 to 10 years, and 6 patients (16.2%) were aged 11 to 14 years. This age grouping was used for exploratory subgroup comparison.

Patient records were reviewed for age, sex, side of involvement, mode of delivery, associated congenital conditions, preoperative findings, operative details, and follow-up data. Patients were included if they were older than 3 years, had not undergone prior surgical treatment for CMT, and had persistent head tilt, cervical motion limitation, and clinically evident SCM tightness. All included patients also had normal ocular and neurological findings and complete clinical follow-up data available for review. Most patients in this series were referred after infancy because of delayed presentation or persistent deformity rather than through a standardized nonoperative treatment pathway at our institution. When available in the retrospective records, prior nonoperative management consisted of physiotherapy and home-based stretching exercises.

Patients with nonmuscular causes of torticollis were excluded. Patients with major associated congenital anomalies or alternative musculoskeletal, neurological, or ocular causes of torticollis were also excluded. All procedures were performed by the same senior surgeon, and the same institutional postoperative rehabilitation protocol was applied throughout the study period.

### 2.3. Preoperative Diagnostic Evaluation

The mode of delivery of the patients was recorded. Ocular and neurological examinations were performed, and anteroposterior and lateral radiographs of the cervical vertebrae were obtained. The presence of additional congenital deformities was investigated. This preoperative evaluation was performed to confirm the diagnosis of CMT and to exclude alternative causes of torticollis, including cervical vertebral anomalies and nonorthopaedic conditions. Surgical treatment was considered in children older than 3 years with persistent head tilt, restricted cervical rotation and/or lateral flexion, and clinically evident SCM tightness.

### 2.4. Surgical Technique

**Figure 1:**
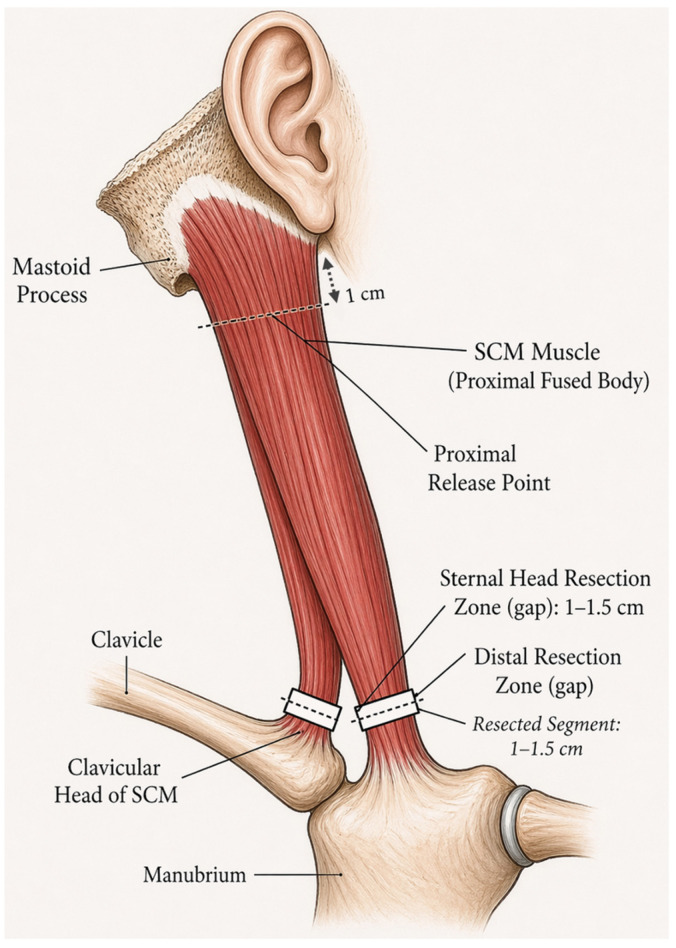
Schematic illustration of the surgical release and resection of the sternocleidomastoid (SCM) muscle. The diagram highlights the proximal release point near the mastoid process and the distal resection zones. A 1 to 1.5 cm segment of both the sternal and clavicular heads is resected distally, creating a clear gap to ensure adequate correction of the contracted muscle and to prevent recurrence.

All procedures were performed under general anesthesia. The patient was positioned supine with the head rotated to the contralateral side to facilitate exposure of the contracted SCM muscle. A skin incision was made one fingerbreadth proximal and parallel to the medial end of the clavicle. The platysma and superficial fascia were incised in line with the skin incision, and the sternal and clavicular attachments of the SCM muscle were then identified. The distal tendon sheath was incised longitudinally to expose the tendon. The jugular vein was identified and preserved, and a right-angle clamp was inserted posterior to the tendon. The tendon was retracted superficially, and straight clamps were placed above and below the right-angle clamp. A 1 to 1.5 cm segment of the tendon was resected distally (Figure 1). Any additional contracted tissues were carefully dissected.

A mini transverse incision was made just inferior to the mastoid process. The tendon borders were exposed using right-angle clamps placed close to the bone. Traction was applied to the tendon through the clavicular incision using a clamp, allowing clear visualization. The tendon was cut close to the bone, beneath the mastoid process, to avoid injury to the accessory nerve. Adequate release was confirmed intraoperatively by correction of head posture and passive improvement in neck motion. The incision sites were closed. The procedure was completed by placing cushions on the operated side to maintain the head in an overcorrected position.

### 2.5. Postoperative Care

For five days postoperatively, patients were kept in the supine position with support placed on the operated side to maintain overcorrection. Subsequently, a physiotherapist initiated manipulative stretching therapy. Patients were instructed to perform active stretching exercises independently, with their parents assisting in passive exercises. They were discharged on postoperative day 7 and advised to perform the exercises 3 times daily for 3 months, with 15 repetitions per session. No cervical brace was used in any patient. The postoperative rehabilitation and in-hospital observation periods reflected the institutional routine and monitoring preferences during the study period. Follow-up visits were scheduled monthly for the first three months, every three months during the first postoperative year, and annually thereafter.

### 2.6. Clinical Assessment

For preoperative and postoperative evaluation, the clinical outcome framework originally described by Lee et al. [22] was used. In the present study, preoperative assessment was limited to the functional components available in the medical records, including neck range of motion, head tilt, and facial asymmetry, whereas postoperative assessment included both functional and cosmetic components, including scar formation, loss of V-column appearance of the sternocleidomastoid muscle, and the presence of lateral bands.

Head rotation and lateral flexion were measured clinically using a goniometer. Limitations of 1 to 10° in rotation or lateral flexion were classified as mild, 11 to 25° as moderate, and more than 25° as severe. Facial asymmetry was documented based on clinical findings, including eye size asymmetry, eyebrow or eyelid drooping, cheek flattening, and unilateral facial hypoplasia. All preoperative and postoperative clinical assessments were based on routine outpatient examinations documented in the medical records. Because of the retrospective design, no separate blinded observer-based cosmetic assessment or patient-reported outcome measure was available. Postoperative nerve function was not evaluated using a formal standardized neurological assessment protocol; therefore, the absence of documented nerve injury was based on routine clinical follow-up records.

The maximum postoperative score was 18. However, because only functional criteria were available for the preoperative evaluation, the maximum preoperative score was 9. According to postoperative total scores, 17 to 18 points were defined as excellent, 15 to 16 points as good, 13 to 14 points as fair, and 12 points or below as poor.

### 2.7. Statistical Analysis

Statistical analysis was performed using IBM SPSS Statistics version 26.0 (IBM Corp., Armonk, NY, USA). Continuous variables were expressed as mean ± standard deviation, and categorical variables as number and percentage. Preoperative and postoperative paired continuous outcomes were compared using a paired-samples *t*-test after assessing the distribution of within-subject differences. The results are reported as mean change with 95% confidence intervals. For subgroup comparisons, the independent-samples *t* test or Mann–Whitney U test was used for continuous variables, and the chi-square test or Fisher’s exact test for categorical variables, as appropriate. A two-tailed *p*-value < 0.05 was considered statistically significant. Age-based subgroup analyses were considered exploratory.

## 3. Results

### 3.1. Patient Characteristics

A total of 37 patients were included in the study. Baseline demographic and clinical characteristics of the cohort are summarized in Table 1.

### 3.2. Preoperative and Postoperative Clinical Outcomes

Preoperative and postoperative clinical outcomes, including subgroup findings by age at surgery, are summarized in Table 2.

**Figure 2:**
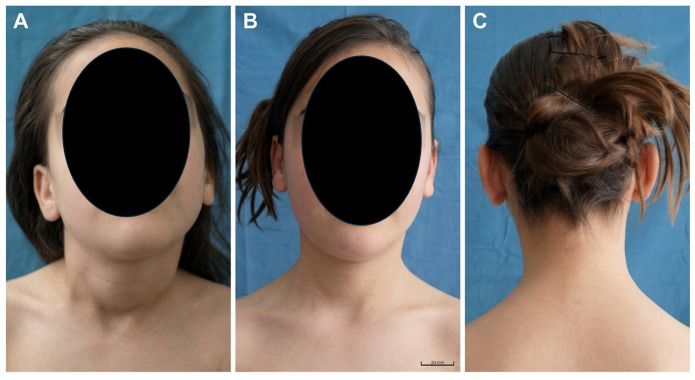
Representative clinical photographs of a child with congenital muscular torticollis treated with distal resection combined with proximal release of the sternocleidomastoid muscle. (**A**) Preoperative frontal view demonstrating head tilt. (**B**) Postoperative frontal view at 1-year follow-up showing correction of head tilt and improved head position. (**C**) Postoperative posterior view at 1-year follow-up demonstrating cervical alignment and neck contour. (Written informed consent for publication of the images was obtained from the patient’s parents or legal guardians.).

At the final follow-up, all major clinical outcomes had improved significantly, with marked postoperative improvement in cervical motion and functional assessment scores (Table 2). No patient had residual head tilt at final follow-up, and most patients achieved excellent or good postoperative outcomes. Mild residual facial asymmetry was uncommon, whereas partial loss of the sternocleidomastoid V-column contour remained a frequent postoperative finding. Representative preoperative and postoperative clinical photographs are shown in Figure 2.

### 3.3. Complications and Revision

Complications were infrequent. Hypertrophic scarring at the clavicular incision site developed in one patient (2.7%). No patient developed lateral band formation, infection, or hematoma. No revision procedure was required, and no recurrence was observed during follow-up. No postoperative neurological deficit was documented in the available routine follow-up records.

### 3.4. Subgroup Analysis According to Age at Surgery

In the exploratory subgroup analysis, postoperative findings were broadly similar between the age groups. Although descriptively excellent outcomes appeared more frequent and residual facial asymmetry was less common in patients aged 3 to 10 years, none of the between-group differences reached statistical significance. V-column contour loss remained common in both groups, and no recurrence was observed.

## 4. Discussion

The main finding of the present study was that distal resection combined with proximal release of the sternocleidomastoid muscle yielded favorable functional and cosmetic results in children older than 3 years with congenital muscular torticollis. At final follow-up, 89.2% of the patients had excellent outcomes, and 10.8% had good outcomes, with marked improvement in cervical range of motion, complete correction of head tilt, no lateral band formation, and no revision surgery. These findings suggest that satisfactory correction can still be achieved in children presenting beyond infancy.

The clinical relevance of our findings becomes clearer when compared with previous reports on distal open release and distal partial resection. Lee et al. [23], reported functional and cosmetic improvement after distal open release in patients aged 6 to 16 years, although some residual deformity-related findings persisted. Similar concerns regarding residual deformity, facial asymmetry, and contour-related issues have also been noted in later studies [3,12]. Likewise, distal partial resection has generally yielded good or excellent results, although postoperative facial asymmetry, contour-related cosmetic concerns, scarring, and residual neck motion limitation have still been reported in some cases [13,22]. More recent studies have also described quality-of-life improvement after surgery and alternative techniques such as endoscopic treatment and unipolar myomectomy [12,17,21]. In contrast, none of our patients developed lateral band formation, residual motion restriction, or recurrent head tilt, and all patients were classified as having either excellent or good results.

Bipolar release has generally been preferred in older children and in more severe deformities because isolated distal release may be inadequate in longstanding contracture [24,25,26]. Wirth et al. [27] reported favorable long-term results after bipolar release, although recurrence still occurred in a small number of patients. Similarly, in another series, full neck motion was not achieved in one patient, and recurrence occurred in one patient despite postoperative bracing [28,29]. These reports suggest that although bipolar release is an established option, it does not completely eliminate the risk of residual deformity or recurrence. Alternative approaches, including tripolar release, have also been described [20]. In our cohort, distal resection combined with proximal release was associated with favorable correction without revision, suggesting that this may be a useful option in selected patients older than 3 years.

Our findings also support the possibility that distal resection may reduce the risk of postoperative reattachment compared with simple distal release. In a study of 83 surgically treated patients with congenital muscular torticollis, postoperative SCM reconnection was observed in 10 patients [30]. Although our study did not include a direct comparison group, the absence of recurrence and revision surgery in our series is consistent with the theoretical benefit of removing a distal tendon segment rather than performing release alone.

The timing of surgery should be interpreted in the context of the established effectiveness of early non-surgical treatment. Recent systematic reviews continue to support physiotherapy and stretching as first-line treatment during infancy, while confirming that surgery remains appropriate in children with persistent deformity after failed conservative treatment or delayed presentation [10,11]. Our cohort reflects this latter scenario, as most patients were referred after infancy because of persistent deformity or delayed presentation, representing late surgical candidates rather than children managed through a standardized early nonoperative pathway.

Another notable finding was the high rate of partial loss of the SCM V-column appearance. Similar findings have been reported after distal partial resection, with SCM column loss observed in 77% to 83% of patients and generally considered cosmetically minor [10,18]. In our series, partial V-column loss was observed in 91.9% of patients. This may represent an expected morphological consequence of distal resection rather than a complication, because removal of a 1 to 1.5 cm tendon segment would be expected to alter the normal muscle contour. Although this finding was frequently documented, it was not recorded as a meaningful cosmetic concern in the available follow-up records. This suggests that restoration of head position and neck motion may be more relevant to perceived success than preservation of cervical contour alone. The weight assigned to isolated V-column loss in postoperative scoring systems may therefore merit further evaluation.

Residual facial asymmetry was uncommon in our cohort and was observed only in older patients. This finding is consistent with previous reports showing that delayed treatment may reduce the likelihood of complete reversal of long-standing craniofacial remodeling, even when functional correction is achieved [12,23,27]. Previous surgical series using different operative techniques have likewise shown that surgery performed after the preschool period can still improve head posture and cervical motion, although residual facial asymmetry or incomplete correction may persist more often in older children [14,18,22,24]. In our series, a similar age-related pattern was observed: residual facial asymmetry was limited to older patients, whereas correction of head tilt and improvement in neck motion were achieved throughout the cohort. Previous surgical series have also separated children older than 10 years from younger pediatric cohorts when evaluating outcomes in congenital muscular torticollis. Cheng and Tang categorized patients as <1 year, 1–3 years, 3–10 years, and >10 years [13], whereas Canale et al. also used a cutoff above 10 years in their long-term evaluation [14]. This age-based distinction appears clinically relevant because younger children may retain greater remodeling potential, whereas older, late-presenting patients are more likely to have established secondary deformity. Our findings support this view and suggest that surgery should not be dismissed solely on the basis of age when persistent deformity, restricted motion, or visible asymmetry remain present.

Another issue is the possible association between longstanding CMT and secondary cervical or cervicothoracic postural adaptation, including compensatory scoliosis. Previous studies have shown that CMT may be accompanied by secondary cervicothoracic scoliosis and long-term postural malalignment, including pelvic malalignment with or without compensatory scoliosis [31,32]. Although cervical scoliosis was not systematically evaluated radiographically in our cohort, this relationship remains clinically relevant because persistent head tilt may affect spinal alignment over time. Future studies with standardized radiographic follow-up may clarify whether correction of SCM contracture improves secondary spinal deformity.

Postoperative management is also relevant. Although halter traction, prolonged cervical bracing, or both have been routinely used after surgery in several published series [18,23,28,29], no cervical brace was used in our cohort. Correction was maintained with early overcorrection positioning, physiotherapist-guided stretching, and a structured home exercise program. The favorable outcomes suggest that routine brace use may not be essential when surgical release is adequate and rehabilitation is closely supervised. However, because rehabilitation strategies were not compared, this interpretation should be treated with caution. Although shorter hospitalization may be feasible in selected contemporary settings [33], the 7-day postoperative stay in our series reflected the institutional protocol used during the study period, which included inpatient overcorrection positioning and supervised initiation of rehabilitation.

### Limitations

This study has several limitations. First, the retrospective single-center design may limit the generalizability of the findings. Because the study was conducted in a specific eastern Mediterranean population, perceptions of cosmetic outcome may differ across sociocultural settings, which may limit the broader applicability of the cosmetic findings. The long study period may also have introduced temporal variation in referral patterns, documentation quality, and follow-up practice. Second, the sample size was relatively small, particularly in the older age subgroup. Third, the absence of a comparison group precluded direct evaluation against other surgical techniques or postoperative rehabilitation protocols. Finally, cosmetic outcomes were assessed clinically rather than with standardized photographic or patient-reported measures, and no blinded observer-based cosmetic evaluation was available. Furthermore, postoperative nerve function was not assessed using a formal standardized neurological protocol, and cervical scoliosis was not analyzed as a predefined study variable.

## 5. Conclusions

In conclusion, distal resection combined with proximal release of the sternocleidomastoid muscle provided favorable functional and cosmetic outcomes in children older than 3 years with congenital muscular torticollis in this retrospective series. The technique was associated with marked improvement in cervical motion, correction of head tilt, low complication rates, and a high proportion of excellent or good results. Postoperative management without routine cervical bracing was successfully applied in this cohort when supported by structured stretching exercises and close follow-up. Although these findings are encouraging, comparative studies are required to determine whether this approach offers measurable advantages over other established surgical techniques.

## Figures and Tables

**Table 1:** Baseline demographic and clinical characteristics of the study cohort.

Variable	Value
Age at surgery, years (mean ± SD; median [IQR]; range)	4.7 ± 2.3; 4.0 [3.0–5.0]; 3–14
Follow-up duration, years (mean ± SD; median [IQR]; range)	3.4 ± 1.7; 3.0 [2.0–4.0]; 1–9
Female sex, *n* (%)	21 (56.8)
Male sex, *n* (%)	16 (43.2)
Right-sided involvement, *n* (%)	23 (62.2)
Left-sided involvement, *n* (%)	14 (37.8)
**Mode of delivery, *n* (%)**	
Normal vaginal delivery	23 (62.2)
Cesarean section	9 (24.3)
Vaginal breech delivery	5 (13.5)
Previous physiotherapy history, *n* (%)	7 (18.9)

Values are presented as mean ± SD, median [IQR], range, or number (%), as appropriate.

**Table 2:** Preoperative and postoperative clinical outcomes of the cohort and postoperative subgroup findings according to age at surgery.

Variable	Preoperative (*n* = 37)	Postoperative (*n* = 37)	Postoperative: 3–10 Years (*n* = 31)	Postoperative: 11–14 Years (*n* = 6)
**Functional outcomes**				
Cervical rotation (°)	54.2 ± 8.6	87.9 ± 3.4 (*p* < 0.001)	—	—
Lateral flexion (°)	24.1 ± 6.8	44.5 ± 3.2 (*p* < 0.001)	—	—
Score-based assessment *	6.8 ± 1.4	14.2 ± 0.9	—	—
**Clinical and cosmetic findings, *n* (%)**				
Head tilt	37 (100)	0 (0)	0 (0)	0 (0)
Facial asymmetry	37 (100)	3 (8.1)	2 (6.4)	1 (16.7)
Partial V-column loss	—	34 (91.9)	28 (90.3)	6 (100)
Hypertrophic scarring	—	1 (2.7)	1 (3.2)	0 (0)
Lateral band formation	—	0 (0)	0 (0)	0 (0)
**Outcome grading, *n* (%)**				
Excellent	—	33 (89.2)	29 (93.5)	4 (66.7)
Good	—	4 (10.8)	2 (6.5)	2 (33.3)

* Preoperative values reflect the functional assessment score based on the available preoperative components, whereas postoperative values reflect the total outcome score including both functional and cosmetic components. Subgroup findings are postoperative and presented descriptively.

## Data Availability

The data presented in this study are available on reasonable request from the corresponding author. The data are not publicly available because they contain information that could compromise patient privacy and are subject to institutional data protection policies.

## Abbreviations

The following abbreviations are used in this manuscript:

CMT	Congenital muscular torticollis
SCM	Sternocleidomastoid
SPSS	Statistical Package for the Social Sciences
SD	Standard deviation
CI	Confidence interval
